# Left and right ventricular strain-volume/area loops: a narrative review of current physiological understanding and potential clinical value

**DOI:** 10.1186/s44156-024-00046-z

**Published:** 2024-05-21

**Authors:** Thijs P Kerstens, Stijn CM Donker, Geert Kleinnibbelink, Arie PJ van Dijk, David Oxborough, Dick H.J. Thijssen

**Affiliations:** 1 Department of Medical BioSciences, Radboud University Medical Center, Geert Grooteplein Zuid 10, 6525 GA Nijmegen, The Netherlands; 2 Research Institute for Sport and Exercise Medicine, Liverpool John Moores University, L3 5UX, Liverpool, United Kingdom; 3 Department of Cardiology, Radboud University Medical Center, Geert Grooteplein Zuid 10, 6525 GA Nijmegen, Netherlands; 4 Department of Medical BioSciences (928), Radboud University Medical Center, 6500HB Nijmegen, P.O. Box 9101, The Netherlands

**Keywords:** Strain-volume loop, Strain-area loop, Speckle tracking echocardiography, Cardiac deformation

## Abstract

Traditionally, echocardiography is used for volumetric measurements to aid in assessment of cardiac function. Multiple echocardiographic-based assessment techniques have been developed, such as Doppler ultrasound and deformation imaging (e.g., peak global longitudinal strain (GLS)), which have shown to be clinically relevant. Volumetric changes across the cardiac cycle can be related to deformation, resulting in the Ventricular Strain-Volume/Area Loop. These Loops allow assessment of the dynamic relationship between longitudinal strain change and volumetric change across both systole and diastole. This integrated approach to both systolic and diastolic function assessment may offer additional information in conjunction with traditional, static, measures of cardiac function or structure. The aim of this review is to summarize our current understanding of the Ventricular Strain-Volume/Area Loop, describe how acute and chronic exposure to hemodynamic stimuli alter Loop characteristics, and, finally, to outline the potential clinical value of these Loops in patients with cardiovascular disease. In summary, several studies observed Loop changes in different hemodynamic loading conditions and various (patho)physiological conditions. The diagnostic and prognostic value, and physiological interpretation remain largely unclear and have been addressed only to a limited extent.

## Background

Several non-invasive imaging modalities are currently available for cardiovascular evaluation, with echocardiography being the most commonly used and accessible method [1]. Traditionally, echocardiography is used for volumetric measurements to aid in assessment of cardiac function, with left ventricular ejection fraction (LVEF) being the most frequently used indicator of left ventricular cardiac function. Multiple additional echocardiography-based measures have been developed, such as Doppler ultrasound and tissue Doppler. Two decades ago, deformation imaging using speckle tracking echocardiography was introduced [1–3]. Deformation imaging enables evaluation of cardiac function by focusing on deformation of the myocardium in different directions (e.g., longitudinal, circumferential, and radial) [4]. A vast number of studies have evaluated systolic strain values, with end-systolic peak global longitudinal strain (GLS) being the most frequently used measure. Several studies have demonstrated GLS to be relevant in detecting cardiovascular abnormalities, whilst GLS has also been associated with cardiovascular events, largely independent of established markers of cardiac function such as LVEF [5].

The dynamic nature of cardiac function and diversity in myocardial fiber orientation may be difficult to assess using isolated measures. Although the traditional pressure volume (PV) loop does offer comprehensive systolic and diastolic measurements irrespective of time, with well-founded implications for systolic *and* diastolic function, this measure does not account for fiber orientation and shortening and its invasive nature has hindered clinical implementation. Myocardial work (representing the area enclosed by the PV loop– i.e., the total force generated by the heart for ejection against afterload) is another comprehensive metric of cardiac function. Multiple studies focused on a non-invasive approach (i.e., the pressure-strain loop) to evaluate myocardial work, but this parameter is predominantly confined to systolic function [6, 7] and is derived from a single peak measure of arterial pressure. The different approaches to assess cardiac function beyond current metrics, emphasize the search for additional ways to assess cardiac function and mechanics.

Fortunately, technological advances facilitate combined measurement of contraction in a specific direction and volumetric changes throughout the cardiac cycle. This led to introduction of the left ventricular (LV) Strain-Volume Loops (SVL) and its right ventricular (RV) equivalent, the Strain-Area Loop (SAL) [8, 9]. It is hypothesized that this measure provides a comprehensive insight into dynamic cardiac function, integrating both systolic *and* diastolic cardiac mechanical/functional characteristics in a non-invasive manner.

Multiple studies have explored how physiological stimuli can acutely and/or chronically alter the SVL/SAL and assessed the Loops in individuals with cardiovascular pathology. In this narrative review, we provide a mechanistic background of the principles underlying the SVL/SAL and outline how these Loops are altered in response to hemodynamic stimuli. Finally, we will summarize the evidence linking the Loops to their potential clinical use in diagnosis or prediction of cardiovascular events and discuss potential future directions.

## How to measure the strain-volume/area loop

Data collection pertaining to strain and volume can be acquired using 2D or 3D echocardiography but is also possible using magnetic resonance imaging (MRI). Data need to be acquired in line with the recommendations for the modality of choice. Although some research has been published based on SVL acquired using MRI and tagging [10], or transesophageal echocardiography [11], this section mainly focusses on acquisition of the Loops using transthoracic (speckle tracking) echocardiography as this technique is more frequently used and more easily available. Below, we have discussed data collection and analysis of strain and volume separately.

*Strain.* Literature on the Loops mostly refers to global longitudinal strain (GLS) when addressing strain. In research towards the SVL, LV GLS was acquired largely in line with recommendations and guidelines for Speckle Tracking Echocardiography, regarding for example frame rate, to enable standardized definitions and acquisition [4, 12]. For the SAL however, despite recommendations to use free wall strain for RV GLS, septal strain is often also included in studies due to a possible role in volume ejection. Although longitudinal mid-wall or myocardial strain is mostly used, endocardial GLS can also be used and studies have also constructed loops based on circumferential, radial (or transverse) strain, or principal strain assessed with 3D echocardiography [8, 13, 14]. Recommendations state to acquire circumferential strain on short-axis slices, but this hampers simultaneous volumetric measurements on 2D echocardiography since the latter is acquired using apical views [4]. Since longitudinal strain is acquired using the apical views, this allows for simultaneous assessment of longitudinal strain and ventricular volume/area. The potential added, synergistic and/or individual value of different directions of strain (i.e., circumferential vs. longitudinal) and layer specific (i.e., endocardial vs. myocardial) strain are currently unknown, and could be a focus for future research studies. Since the current state of our knowledge is primarily based on longitudinal strain, we therefore primarily focus on Loops derived based on GLS.

*Volume/Area.* Cardiac volumetric data, both for the RV and LV, can be acquired in line with recommendations for cardiac chamber quantification [12]. For these purposes, different combinations of views can be used. For LV volumetric assessment, it is recommended to use a combination of apical 2- and 4- chamber view to limit geometrical assumptions [12]. In current literature regarding the Loops, LV volume has been assessed on a variety of echocardiographic views, e.g. using only the apical 4-chamber view [9], a combination of the apical 2, 3, and 4 chamber view [15], and with 3D echocardiography [8, 14]. RV dimensions and Fractional Area Change (FAC) are best estimated using a RV focused apical 4-chamber view and is expressed in area (cm^2^) instead of volume (mL) due to its complex geometry [12].

*Construction of the Loop.* Different methods are described to construct the Loops and acquire its related characteristics. There is not one single standardized method for constructing Loops in literature so far. Depending on the modality (2D vs. 3D echocardiography, MRI) and vendor, strain and volume/area can be acquired simultaneously or separately. Separately acquired strain and volume/area data require additional steps to match timing in the cardiac cycle (e.g., spline interpolation). Due to the different methods, SVL/SAL construction is mostly tailored for a specific setting or specific software used to acquire strain and volume data, currently hampering inter-vendor usability. Most often, Loops are constructed across one cardiac cycle. A variety of in-house developed solutions have been described in literature, ranging from structured templates in Microsoft Excel to vendor-specific scripts (e.g., MATLAB for TomTec).

## What characteristics do the loops have?

**Fig. 1:**
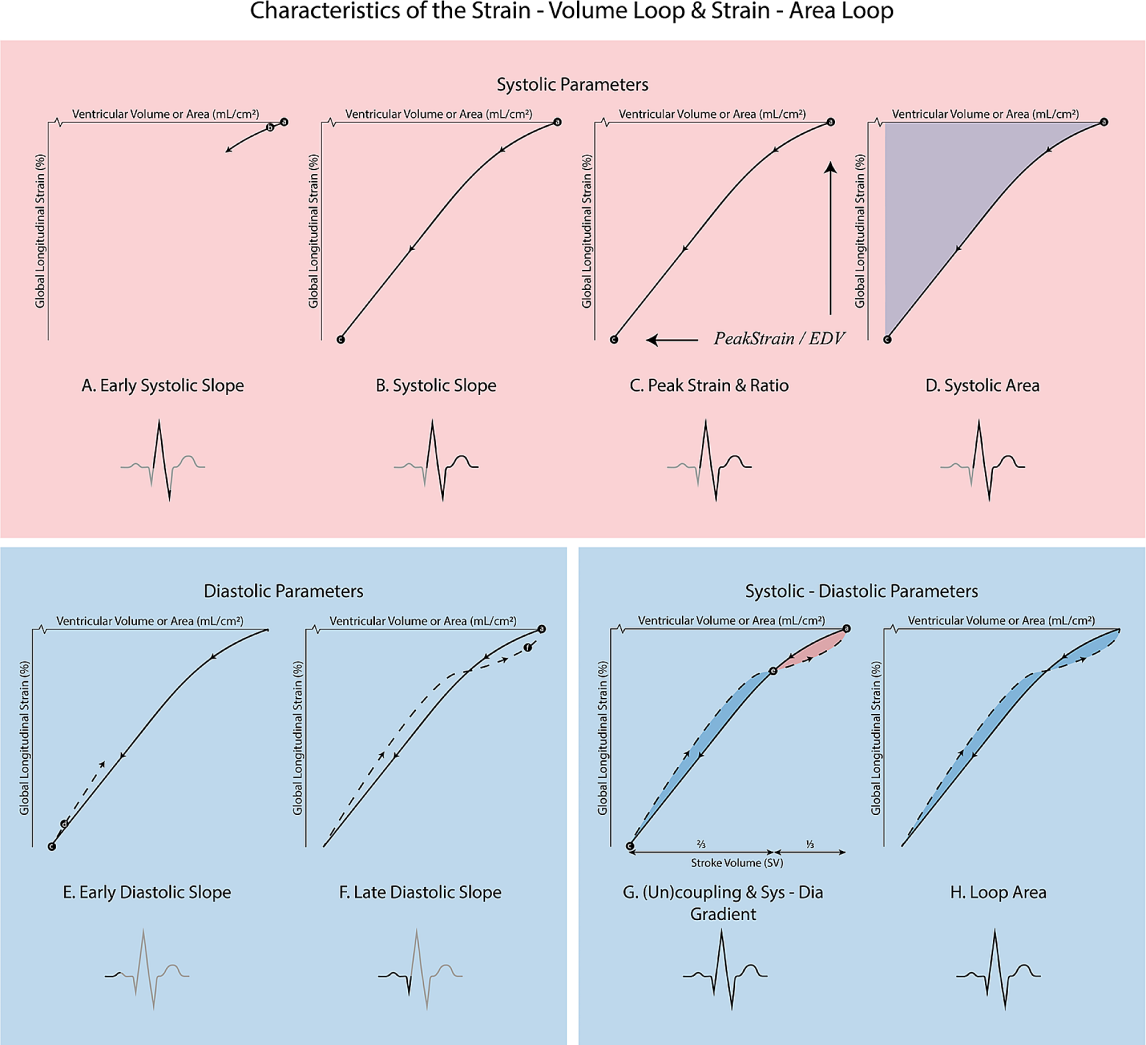
Strain– Volume/Area Loop characteristics used in literature to describe the dynamic interplay between longitudinal strain and volume/area of both the left and right ventricle of the heart. Characteristics presented chronologically. Shape of presented Loop is merely chosen for visualization purposes

The SVL/SAL provides insight in the relation of global longitudinal strain and volume/area across the cardiac cycle, which is not captured by other techniques. However, it is important to note that the actual shape and deformation in other directions (e.g., circumferential strain) contributing to volume change are unknown. The Loops do not weigh these factors contributing to volume change, as illustrated by a mathematical model [16]. The shape of the relation between GLS and volume/area across the cardiac cycle can be captured using multiple parameters, largely comparable for both the LV and RV. These parameters are mostly arbitrarily chosen, and some calculation methods lack strong scientific foundation (e.g., early systolic slope based on the first 5% of stroke volume). Common parameters (e.g., peak strain) are included as well as parameters that are obtained specifically through constructing the Loop (e.g., slopes). The Loop parameters are discussed below, in chronological order of appearance across the cardiac cycle starting at systole. Figure 1 provides a multi-panel overview of these parameters.

### Systole

- *Early systolic slope*: represents a least-squares fit slope of the relation between strain and volume during the ejection of the first 5% of stroke volume (SV), starting at the beginning of systole [17].
- *Systolic slope*: represents the slope of the relation between strain and volume starting at end-diastole up to end-systole [8].
- *Systolic area*: the area between the x-axis and the systolic line of the SVL [11].
- *Peak strain*: maximum deviation from y-axis (i.e., strain). This negative value is defined as the end-systolic longitudinal strain. In line with recommendations, references to strain increase or decrease apply to the absolute value (i.e.,|x|) of the actual peak strain [4]. Related to this characteristic, an additional ratio has been reported, represented as peak strain divided by end-diastolic volume [8].

### Diastole

- *Early diastolic slope*: slope of the strain– volume relationship during the first 5% of ventricular filling in diastole, starting at end-systole [17, 18].
- *Late diastolic slope*: slope of the strain– volume relationship during the final 5% of increment of ventricular filling, i.e., up to end diastolic volume [17, 18].

### Systolic - Diastolic

- *(Un)coupling (also sys-dia gradient)*: descriptive parameter representing the difference in GLS when measured at a fixed ventricular volume during systole compared to diastole (Fig. 1). It has been quantified using various methods. Uncoupling needs not to be confused with ventriculo-arterial coupling (slope of the end-systolic pressure volume relation (PVR) in relation to the end-diastolic PVR in a traditional PV loop, which is indicative of the interaction between the heart and arteries). Lord et al. described systolic-diastolic strain gradients, i.e., differences between systolic and diastolic strain for pre-defined percentages (10% intervals) of end diastolic volume [19]. Later studies adopted a similar measure, i.e., uncoupling, representing the average difference in systolic and diastolic strain (systolic strain *minus* diastolic strain) for any given volume across the cardiac cycle [17]. Uncoupling indicates that there is a difference in the strain-volume relation in systole versus diastole. Although a precise physiological explanation is lacking, changes in uncoupling seem to indicate changes in cardiac mechanics or cardiac function. Uncoupling has also been arbitrarily divided in early and late diastolic uncoupling. This is based on a cut-off of the first 2/3 of volume increase (early diastolic uncoupling) and the subsequent 1/3 volume increase (late diastolic uncoupling). Due to variable contribution of atrial contraction to ventricular filling, this subdivision cannot be used to distinguish between early filling and atrial contraction [20].
- *Coefficient of determination (R*^*2*^*– S/D coupling)*: coefficient of determination of one linear fitting curve for the Strain-Volume Loop across the cardiac cycle including both systolic and diastolic components; a higher value represents more similar strain values during systole and diastole across the Loop [14].
- *Loop area*: the area enclosed by the plotted Loop, based on the area between the systolic strain– volume relation and diastolic strain– volume relation using the rectangle method [21].

### Reproducibility

Multiple studies demonstrated good-to-excellent reproducibility of strain (i.e., typically end-systolic peak) and volumetric (i.e., typically end-systolic and end-diastolic) measurements [12, 22], including at specific percentages of the cardiac cycle [9]. SVL characteristics derived from the apical 4-chamber view showed moderate-to-excellent intra-rater agreement for early systolic slope (ICC = 0.95), Systolic slope (ICC = 0.95), peak strain (ICC = 0.83), early diastolic (ICC = 0.78) and late diastolic (ICC = 0.74) uncoupling [17]. The systolic–diastolic gradient showed poor reproducibility for the LV and poor-to-good reproducibility for the RV [9]. Regarding the SAL, moderate-to-excellent intra-rater agreement for (early) systolic slope, peak strain, (early and late diastolic) uncoupling, early and late diastolic slope [23–25]. 3D-echocardiography showed good-to-excellent reproducibility for the slope of the LV Loop and systolic area [11]. To conclude, these studies showed that most characteristics of the Loops show good intra-rater agreement (moderate-to-excellent) and could be improved through standardization of acquisition parameters, such as frame rate. However, for some Loop characteristics reproducibility has not been studied to our knowledge (e.g., loop area, LV early and late diastolic slope). It is important to note that reproducibility has mostly been assessed for Loops based on monoplane (apical 4-chamber) echocardiography, thus lacking data on triplane echocardiography. In general, the inter-rater variability and test-retest variability of the Loops are currently unknown. These are important aspects to facilitate translation to clinical practice.

## Loop responses following changes in hemodynamic stimuli

### Left ventricular strain-volume loop: preload and afterload

**Fig. 2:**
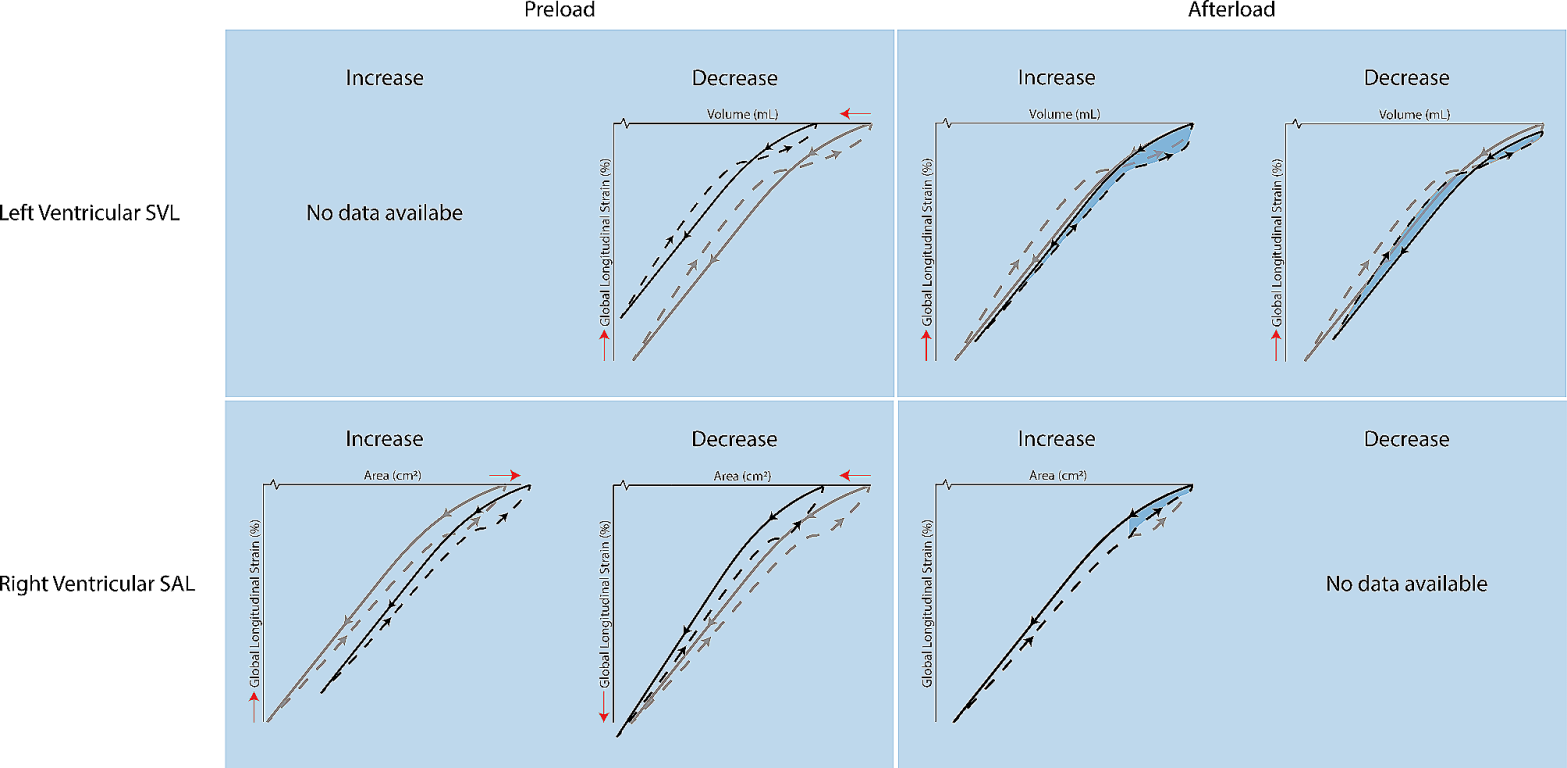
Summary of Strain– Volume/Area Loop changes in response to preload and afterload manipulation. Colors represent changes compared to baseline. Leftward or rightward shift is denoted by horizontal arrow on x-axis. Change in peak strain is denoted by vertical arrow on y-axis. Change in shape is denoted by colored patches. Shape of presented SVL/SAL is merely chosen for visualization purposes and based on previous literature

Various studies have assessed SVL following manipulating cardiac hemodynamics, specifically preload and afterload. A summary of changes in Loop characteristics following manipulation can be found in Fig. 2. For example, a *reduction* in preload was provoked through performing a head-up tilt test in healthy males, which causes an immediate redistribution of blood in the body, leading to a decreased venous return [13]. Consequently, this resulted in a leftward shift of the Loop, indicating a reduced end diastolic volume. In addition, a decrease in longitudinal strain was found with a concomitant increase in transverse strain. Other SVL characteristics were not assessed. No other studies examined SVL characteristics related to changes in preload.

Afterload seems associated with specific Loop characteristics. To study these effects, an anti-gravity suit was used. This is a garment fitted with inflatable bladders, capable of applying external pressure on the lower extremities and abdomen. Through inflation, afterload can be increased. For example, inflation of an anti-gravity suit leads to a decrease in peak strain, a lower early diastolic slope, higher late diastolic slope, and an increase in uncoupling in both young and older men, potentially due to increased afterload [18]. However, it is important to emphasize that inflation of the antigravity suits may have also simultaneously increased preload [26]. Interestingly, another study examined patients with severe aortic valve stenosis following aortic valve replacement (AVR), which resulted in a *decrease* in afterload [27]. Opposite to the observations in healthy younger and older individuals using the anti-gravity suit following an increase in afterload, the decrease in afterload after AVR caused a lower uncoupling. Peak strain was lower post-AVR, which seems contradictory given that peak strain also decreased after increasing afterload in healthy subjects. However, it should be highlighted that antigravity suit inflation and cardiac surgery occurred in healthy and diseased subjects, respectively. The lower peak strain in the diseased subjects was assessed shortly after cardiac surgery (i.e., post-AVR). Although the physiological mechanism of these SVL changes remain unclear, Loop characteristics seem responsive to afterload manipulation.

In line with short-term changes after cardiac surgery for aortic valve stenosis, the SVL was subject to change after a median follow-up of 1.25 years [27]. Specifically, an increase in peak strain was observed (post-AVR: -14.2 ± 4.0; follow-up: -16.9 ± 0.16; *p* < 0.001). A moderate correlation between post-AVR change in uncoupling and change in LV mass was observed (*r* = 0.407 and *r* = 0.439; *p* < 0.05 for late diastolic and total uncoupling, respectively). Most patients with decreased uncoupling (i.e., post-AVR compared to pre-AVR) showed a reduction in LV mass during follow-up. Interestingly, post-AVR valve characteristics were not correlated with subsequent LV remodeling. These data suggest that early changes in cardiac mechanics identified using the Loop characteristics are related to long-term remodeling, i.e., LV mass regression within this patient population.

### Right ventricular strain-area loop: preload and afterload

Kleinnibbelink et al. examined changes of the SAL in response to hypoxia, which causes an increase in pulmonary vascular resistance (PVR; and therefore, an increase in afterload). In hypoxia (i.e., fraction of inspired oxygen [FiO_2_] 14.5%), the SAL showed less uncoupling (not to be confused with ventriculo-arterial coupling) in late diastole and a trend towards a less steep systolic slope [24]. These observations are in line with a study in patients with PAH, where peak strain and uncoupling decreased contingently with increasing PVR under resting conditions [28]. This suggests that Loop characteristics are responsive to changes in PVR, i.e., afterload.

Another study investigated SAL characteristics following preload manipulation [29]. In seven individuals who underwent right heart catheterization, preload was manipulated through intravenous saline infusion (i.e., increasing preload) and inferior vena cava balloon inflation (i.e., decreasing preload). Increase in preload resulted in a rightward shift and a lesser systolic slope, whereas a decrease in preload had the opposite effect on the SAL. Moreover, a significant correlation (*r* = 0.98) was observed between slopes of the invasive end-systolic pressure-area relation (i.e., gold-standard cardiac contractility measure) and slopes of the non-invasive end-systolic strain-area relation (i.e., as measured by SAL). This suggests that the Loops might provide a valuable estimate of cardiac contractility, although the small sample size must be considered.

Taken together, these studies indicate that, both LV and RV Loop characteristics show changes in response to different hemodynamic stimuli (Fig. 2). Unfortunately, since responses may differ between healthy and diseased, and considering the difficulty in manipulating merely a single factor (i.e., preload or afterload), further research is required to better understand the exact changes and responses.

### Left ventricular strain-volume loop: exercise bout

Studies have also used the SVL to assess changes in cardiac mechanics upon a bout of exercise. Lord et al. observed a leftward shift of the Loop, combined with a lower peak strain and a change in systolic– diastolic gradient (at 40%, 70% and 80% of end-diastolic volume) of the SVL in fifteen participants after a 100-mile ultramarathon. The leftward shift is possibly a consequence of changes in preload, afterload, and contractility as well as concomitant RV dilation affecting LV volume following exercise (i.e., interventricular dependence). These latter volumetric changes have been reported previously in the literature [30]. Interestingly, this post-exercise leftward shift seems to match earlier observations following preload manipulation [9, 13]. During recovery (6 h post-exercise), the SVL systolic– diastolic gradient returned towards baseline, whilst the leftward shift remained present.

### Right ventricular strain-area loop: exercise bout

Lord et al. also examined the impact of a 100-mile ultramarathon on the RV and observed a rightward shift of the SAL (i.e., RV dilatation) [9]. RV dilatation post-exercise may be caused by RV volume and pressure overload following endurance exercise. Previous studies have described interventricular septum displacement in response to RV pressure/volume overload, which aligns with the observed rightward shift of the SAL (and also the abovementioned leftward shift of the SVL) [31]. Despite these volumetric changes, no change in systolic–diastolic gradient following prolonged exercise was found. In marked contrast, Kleinnibbelink et al. examined 45-minute bout of high-intensity running exercise in 21 healthy individuals and showed no rightward shift of SAL, despite presence of reduction in peak strain and uncoupling [24]. These findings are consistent with other studies of high-intensity short duration exercise [32, 33].

In summary, these studies show that the SAL and SVL are altered in response to exercise-induced hemodynamic changes in loading condition (i.e., preload and afterload) or changes in intrinsic contractility/relaxation upon exercise. Since cardiac function assessment using the Loops (and their relation to hemodynamic stimuli) in health and disease is not completely understood, more research could elaborate on this.

### Left ventricular strain-volume loop: exercise training

Multiple studies investigated the SVL in relation to exercise training, and explored whether training alters Loop characteristics. In a cross-sectional study, Oxborough et al. revealed a higher peak strain in elite athletes with *low static-low dynamic* and *high static-high dynamic* exercise compared to athletes with either *low static-high dynamic* or *high static-low dynamic* exercise [19]. Additionally, higher strain values were observed both in systole and diastole for several LV volumes in athletes with *high static-high dynamic* exercise. Regardless, no differences in uncoupling between groups were observed. The increased strain during systole and diastole was weakly correlated with LV mean wall thickness (*r* = 0.25-0.41 and *r* = 0.37, respectively) and authors suggest that differences in strain across the cardiac cycle could be partly driven through geometric adaptations.

In Olympic rowers (*n* = 27, 19 males) increase in wall thickness, diameter, volume, and mass were observed after 9 months training, whilst female rowers showed larger (absolute and indexed to body surface area) adaptation in LV diameter and mass [34]. Despite these adaptations, no changes in LV Loop characteristics were found following exercise training. Interestingly, female rowers showed steeper systolic and late diastolic slopes compared to males. However, these steeper slopes may be, at least partly, explained by the smaller unscaled LV volume in female rowers. Another study showed an increase in LV mass without changes in peak strain in 23 young healthy male subjects receiving 24-week endurance (*n* = 10) and resistance training (*n* = 13) [35]. In both groups a slight rightward shift of the Loop was observed, in the absence of significant change in LV end diastolic volume. Systolic– diastolic gradients (related to uncoupling) showed no changes following training. Taken together, these studies suggest that regular exercise training may alter LV volumetric and mass characteristics, with limited changes in Loop characteristics in these elite athletes.

### Right ventricular strain-area loop: exercise training

Research also investigated the effects of chronic hemodynamic stimuli on the SAL. In a cross-sectional study, Oxborough et al. found that RV peak strain was not different between athletes from different exercise modalities (i.e., low/high statics/dynamics). However, athletes engaging in *high static-high dynamic* exercise showed ventricular enlargement and a trend towards lower strain during systole compared to some, but not all other exercise modalities. These findings are in line with previous research suggesting lower RV strain in athletes with larger ventricles [19, 36]. Comparatively, the SAL visually seemed to exhibit more uncoupling than the SVL, possibly attributable to distinct filling mechanisms. In another study, no structural and functional adaptation was found after a 9-month increase in training volume (i.e., from 24 to 30–35 h weekly) in elite rowers [34]. There were no differences in RV structural adaptation between sexes. Although some differences between males and females were found in the Loops (a higher systolic slope, early systolic slope, and late diastolic slope in females), these differences were not altered by training and could relate to sex-specific mechanics or smaller RV dimensions in females. To explain the absence of loop changes upon training intensification, the authors suggest a physiological plateau in RV adaptation, which the elite athletes may have already reached before training intensification. Previous literature also suggested existence of a plateau in RV adaptation, though athlete aging and sport diversity posed limitations [37]. Another study, in a less trained (< 2 h exercise/week) population, showed right ventricular and atrial enlargement following 12 weeks of hypoxic (FiO_2_ 14.5%) running exercise. For RV function, only RV fractional area change increased. The SAL exhibited a rightward shift, less uncoupling and lesser systolic and diastolic slopes. Although interpretation is difficult, these changes could fit enlarged cavities (especially the rightward shift and decreased lopes [25]). In summary, these studies may indicate that RV dimensions affect strain, and that frequent exercise can lead to RV enlargement, with potential stabilization over time. Similar to the SVL response to exercise training, long term exercise was accompanied by little change in SAL characteristics in some studies, where this might be due to concomitant factors such as an RV remodeling plateau in athletes. However, exercise training in less trained individuals, lower strain and decreased uncoupling was reported.

## Loops as an indicator of cardiovascular disease and adverse events

### Left ventricular strain-volume loop– pathology

Several studies assessed the SVL in relation to different pathologies. Firstly, Lilli et al. compared SVL characteristics between different groups, i.e., healthy subjects, after acute myocardial infarction with normal LVEF, ischemic cardiomyopathy with reduced LVEF, and hypertrophic/infiltrative cardiomyopathy [8]. Patients with previous myocardial infarction but normal LVEF, and patients with ischemic cardiomyopathy with reduced LVEF showed lower systolic slopes and lower strain to end-diastolic volume ratios compared to healthy controls. Authors suggest the lower slopes are likely a consequence of increased LV volume and lower strain. In contrast, patients with hypertrophic or infiltrative cardiomyopathy showed no significantly different slopes and ratios compared to controls. Although other Loop characteristics are not assessed, the results suggest different cardiac mechanics in various pathologies, possibly related to eccentric and concentric remodeling in relation to these pathologies.

Hulshof et al. examined the SVL in patients with severe aortic valve disease, i.e., stenosis and regurgitation. In addition to a lower peak strain, Hulshof et al. observed lower early systolic slope and systolic slope, whilst uncoupling was higher in patients with severe aortic valve disease compared to controls [17]. Importantly, these Loop differences were observed in the presence of a preserved LVEF, suggesting the potential for Loop characteristics to provide additional insight in the dynamic consequences of valvular pathology. Similarly, work by Zhu et al., suggested that SVL could provide potentially valuable information regarding cardiac responses following cardiac resynchronization therapy (CRT) [14]. Forty heart failure (HF) patients scheduled for CRT underwent 3D echocardiography and showed lower slopes and a lower coefficient of determination (R^2^– S/D coupling) of the linear fitting curve compared to healthy controls. Furthermore, a lower coefficient of determination, which represents a measure of (un)coupling, was associated with a beneficial response to CRT defined as a reduction in LV end systolic volume ≥15% at 6-month follow-up.

In recent years, studies have also increasingly focused on (surrogates) of diastolic function and the SVL. First, patients with amyloidosis (n = 25) and Heart Failure with preserved Ejection Fraction (HFpEF) (n = 25) showed smaller and mid-range global area of the Loop (i.e., loop area) compared to healthy controls. In addition, altered classical indices of diastolic function (e.g., greater left atrial volume index, greater mitral average E/e’ ratio) were observed [21]. These data provide first evidence that Loop characteristics may be altered in subjects with diastolic dysfunction. In line with these results, Kerstens *et al*. observed a higher uncoupling of the Loop to be associated with the presence of diastolic dysfunction (according to the ASE/EACVI 2016 recommendations [38]) in patients with suspected HFpEF (*n* = 151) [15]. These findings seem contradictory; however, these two characteristics are difficult to compare. The global loop area is influenced by stroke volume [39], whilst uncoupling (i.e., an average difference between the systolic and diastolic part) is not. Altogether, this suggests that uncoupling of the Loop seems related to the presence of diastolic dysfunction. However, the association, and particularly the discriminative value, of the SVL in relation to diastolic function assessed using invasive pressure measurements is of interest to assess its diagnostic value in clinical practice.

### Right ventricular strain-area loop– pathology

Limited research explored SAL characteristics to cardiovascular conditions. One study by Hulshof et al. explored differences in SAL between pulmonary arterial hypertension (PAH) patients (*n* = 42) *versus* healthy controls [28]. A rightward shift, possibly a consequence of pressure overload, was observed in patients with PAH. In addition, PAH patients also demonstrated a lower peak strain and decreased uncoupling (potentially due to increased longitudinal diastolic relaxation to attenuate for diastolic dysfunction in PAH). Additionally, PAH patients were categorized based on PVR to relate SAL characteristics to PVR levels. The rightward shift and changes in peak strain were contingent upon increasing PVR. Furthermore, Hulshof et al. showed a good discriminative capacity of systolic slope and peak strain for PAH *versus* healthy controls (area under the curve (AUC) of receiving operating characteristics (ROC) > 0.80). In addition, for discriminating between higher (> 500 dynes/sec/cm^5^) *versus* lower (< 500 dynes/sec/cm^5^) PVR levels in PAH, the systolic slope and peak strain even showed superiority compared to some standard echocardiographic indices. Considering the minimal publications on the topic, these interesting results warrant further research to better understand SAL characteristics in cardiovascular pathology.

### Left ventricular strain-volume loop– adverse events

To our knowledge, only one study focused on the association of the SVL and adverse events. Kerstens et al. examined the association of SVL at baseline in 253 individuals with HFpEF and adverse events across median follow-up of 2.9 years [40]. They observed a non-linear association for the ED slope and adverse events (i.e., a composite of all-cause mortality and heart failure related hospitalization). Interestingly, measures of systolic function (e.g., peak strain) were not associated with adverse events in this study. This observation highlights the potential of SVL, but more studies are required to understand the value of SVL characteristics alongside conventional echocardiographic measurements for example in predicting future clinical events.

### Right ventricular strain-area loop– adverse events

At the date of this review, a single study addressed the association of SAL and adverse cardiovascular outcomes. Hulshof et al. focused on the relation between SAL characteristics and all-cause mortality across a 5-year follow-up period of pre-capillary pulmonary hypertension (PH) patients (*n* = 143) [23]. The authors used ROCs to define cut-off values for SAL characteristics to distinguish between alive and deceased patients. In a multivariable analysis, peak strain lower than the cut-off of 14.45% was significantly associated with higher risk for all-cause mortality. Besides peak strain, other individual Loop characteristics were not associated with all-cause mortality. Categorizing Loops based on the cut-off values as “low risk” (i.e. 0–3 high-risk Loop characteristics) and “high risk” (i.e. 4–6 high-risk Loop characteristics) suggested enhanced risk stratification for patients classified as “high-risk” according to international guidelines for diagnosis and treatment of PH [41]. These results emphasize the potential benefit of combining multiple Loop characteristics to improve current methods for risk stratification in pre-capillary PH patients.

## Conclusion

In conclusion, several studies have examined and observed that immediate changes in hemodynamic loading conditions, i.e., preload and afterload, result in changes in cardiac mechanics that can be detected using the Strain-Volume/Area Loops. In addition to changes in traditional echocardiographic measures (volume, peak strain), studies reported changes in diastolic and systolic strain-volume slopes and/or (un)coupling of the Loops. Although interpretation of the results is challenging, these studies suggest that Loop characteristics are responsive of changes in cardiac hemodynamics. However, since most studies utilizing the Loops are in an explorative setting and only a limited number of studies have been published within specific clinical areas, head-to-head comparison to currently available techniques for cardiac assessment remains a point of interest in order to determine their added clinical value.

## Future developments

Loop characteristics seem linked to long-term cardiac adaptations in physiological (e.g., exercise training) and pathophysiological conditions (i.e., disease). Different cardiac pathologies, both affecting the left (e.g., aortic valve disease, HFpEF) and right ventricle (e.g., PH), result in changed Loop characteristics. These Loop changes suggest that cardiac mechanical alterations go beyond merely a shift of the Loop due to changes in ventricular dimensions. However, its discriminative value, and thus diagnostic potential, seem currently limited and require more research. Moreover, the feasibility and utility of including the SVL/SAL into clinical practice needs to be investigated, and addressing intra- and inter-observer variability is essential to ensure reliability and reproducibility of Loop parameters. Across multiple studies, altered uncoupling (or its equivalents, such as global area and R^2^ - sys/dia) of the systolic *versus* diastolic strain - volume relation was observed. Details on the possible underlying mechanisms and implication for cardiac function of uncoupling, and whether this is pathognomonic for specific conditions, remains unclear.

Some studies addressed the association of SVL/SAL characteristics and clinical events during follow-up in subjects with cardiovascular disease (e.g., PH, HFpEF). The results of these early studies warrant future work to better understand the clinical interpretation and prognostic (predictive) value in conjunction and in comparison with conventional measures. Although semi-automated tools exist for SVL/SAL construction [15, 21], standardization and integration of these tools in the clinical workflow has not yet been achieved. It is important to highlight the potential of other modalities (e.g., MRI [10]) and Artificial Intelligence for both image analysis and Loop analysis (e.g., machine learning approaches). Currently, the loops are expressed using isolated predefined parameters, whereas novel parameters potentially exist and artificial intelligence might provide opportunities to distil Loop characteristics. A one-size-fits all approach unlikely represents the holy grail, i.e., (patho)physiological interpretation might require simple and interpretable characteristics, whilst optimal prediction and classification might benefit from complex and combined measurements.

## Data Availability

Data sharing is not applicable to this article as no datasets were generated or analysed during the current study.
